# Functional Analysis of the Cyclin E Gene in the Reproductive Development of Rainbow Trout (*Oncorhynchus mykiss*)

**DOI:** 10.3390/biology14070862

**Published:** 2025-07-16

**Authors:** Enhui Liu, Haixia Song, Wei Gu, Gaochao Wang, Peng Fan, Kaibo Ge, Yunchao Sun, Datian Li, Gefeng Xu, Tianqing Huang

**Affiliations:** 1 Heilongjiang River Fisheries Research Institute, Chinese Academy of Fishery Sciences, Harbin 150070, China; liuenhui@hrfri.ac.cn (E.L.); songhx1998@163.com (H.S.); guwei@hrfri.ac.cn (W.G.); gaochaowang@ymail.com (G.W.); fanpeng@hrfri.ac.cn (P.F.); gekaibo@hrfri.ac.cn (K.G.); sunyunchao@hrfri.ac.cn (Y.S.); lidatian@hrfri.ac.cn (D.L.); 2 State Key Laboratory of Mariculture Biobreeding and Sustainable Goods, Harbin 150070, China; 3 College of Fisheries and Life Sciences, Dalian Ocean University, Dalian 116011, China

**Keywords:** rainbow trout, *Cyclin E*, meiosis, reproduction

## Abstract

As a commercially valuable aquaculture species, rainbow trout (*Oncorhynchus mykiss*) urgently require solutions to growth inhibition associated with reproductive development. To elucidate the function of the cell cycle regulator Cyclin E genes (*CCNE1* and *CCNE2*) in this process, we cloned the genes and analyzed their relative expression across various tissues and gonadal developmental stages. Using RNA interference (RNAi) and overexpression in RTG2 cells, we examined the effects of *CCNE* on cell viability, proliferation, and meiotic gene expression. Results showed that the open reading frame lengths of *CCNE1* and *CCNE2* were 1230 bp and 1188 bp, encoding 408 and 395 amino acids, respectively. Both proteins contain two conserved cyclin boxes, exhibit high structural similarity, and are phylogenetically most closely related to *Oncorhynchus tshawytscha* and *Oncorhynchus kisutch*. Expression and localization analyses revealed that *CCNE1* was highly expressed in the ovary, while *CCNE2* was highly expressed in the testis. Both proteins were expressed during fertilized egg development and key gonadal stages (at 13, 21, and 35 months post-fertilization). *CCNE* expression positively correlated with RTG2 cell viability and proliferation, with immunofluorescence confirming that CCNE is localized in the nucleus. Knockdown or overexpression of CCNE induced the differential expression of reproductive-related genes and key meiotic regulators. These findings suggest that *CCNE1* and *CCNE2* balance meiosis and gamete development through specific regulatory mechanisms, and their dysregulation may be a key factor underlying meiosis inhibition and reproductive development abnormalities.

## Simple Summary

Rainbow trout is an economically important aquaculture species. However, gonadal development will cause growth retardation and reduced muscle quality, leading to significant economic losses. Cyclin E, a crucial regulator of cell growth, proliferation, and meiosis (influencing homologous chromosome pairing, synapsis, and DNA repair), is also vital for reproductive development. This study investigated the functional roles of two cyclin E subtypes (*CCNE1* and *CCNE2*) in regulating rainbow trout reproduction using RNAi and overexpression experiments. Our results indicate that spermatogenesis primarily relies on a *CCNE2*-dominated pathway, while oogenesis utilizes both *CCNE1* and *CCNE2* concurrently. Both subtypes affect gamete development by regulating key meiotic genes. This research provides valuable insights into the role of cell cycle genes in fish reproduction regulation.

## 1. Introduction

The cell cycle, defined as the period from the completion of one mitotic division to the conclusion of the next, is tightly regulated by cyclins, cyclin-dependent kinases (CDKs), and cyclin-dependent kinase inhibitors (CKIs) [1,2,3]. *Cyclin E* (*CCNE*) was first identified in 1991 through screening of a human cDNA library screen [4,5]. This gene is involved in cellular functions such as chromatin pre-replication complex assembly and megakaryocyte polyploidization, playing a key role in coordinating cell division and growth and maintaining normal cell cycle progression [6]. The *CCNE*-*cdk2* complex is essential for the G1/S phase transition, though a minor fraction of *CCNE* also interacts with alternative CDKs, including *cdk1* (formerly *cdc2*) [7]. In higher eukaryotic organisms, *CCNE-cdk2* complexes contribute significantly to DNA replication initiation [8,9]. CCNE overexpression accelerates G1 progression, initiates DNA synthesis, reduces cell size, and diminishes growth factor dependence [10]. Most species possess two isoforms, Cyclin E1 (CCNE1) and Cyclin E2 (CCNE2), both of which are expressed during the cell cycle [4].

Cyclin E has an essential role in the development of several different organisms, such as the black tiger shrimp (*Penaeus monodon*) [11], the Pacific oyster (*Crassostrea gigas*) [12], and the ridge-tailed white prawn (*Exopalaemon carinicauda*) [13]. *CCNE* is inferred to play a regulatory role in the ovary and is involved in nutrient accumulation and transport [14]. Dai et al. reported that the *CCNE-cdk2* complex was highly expressed in the ovaries of *Penaeus vannamei*, indicating its critical involvement in ovarian maturation and oogenesis [11]. In testis, the mRNAs of both *CCNE* isoforms are expressed in spermatogonia, with CCNE2 protein levels exceeding those of CCNE1. *CCNE-*deficient spermatocytes in mammals exhibit abnormalities in chromosome pairing, synapsis formation, DNA double-strand break repair, and telomere stability maintenance [15]. Although the role of cyclin has been widely studied, its function in fish reproductive development is still poorly understood.

Rainbow trout (*Oncorhynchus mykiss*) is an important economic fish in aquaculture. According to the 2024 China Fisheries Statistics Yearbook, the annual domestic production of rainbow trout in 2023 was 41,116 tons [16]. In recent years, the use of chromosome group breeding in triploid rainbow trout has solved the problem of growth inhibition due to sexual precocity in diploids and shortened the breeding cycle of rainbow trout, generating more economic value [17]. Triploid sterility also provides a valuable model for studying fish fertility regulation mechanisms. Our previous transcriptome analysis of diploid fertile and triploid sterile rainbow trout ovarian tissues revealed differential expression patterns of *CCNE1* and *CCNE2* across developmental stages, indicating their important regulatory roles in meiotic repression [18]. However, their precise regulatory functions remain unclear. Therefore, we proposed to investigate the effects of the knockdown and overexpression of key cell cycle regulatory genes on the proliferation and viability of rainbow trout, as well as on meiotic progression and reproductive development at both organismal and cellular levels. This will elucidate the specific role of CCNE in regulating fertility.

In this study, we cloned the *CCNE1* and *CCNE2* genes of rainbow trout and conducted a bioinformatics analysis. We investigated their expression patterns across various tissues and different times of gonadal development. Immunohistochemical localization further revealed stage-specific protein distribution patterns throughout key developmental periods. To elucidate the specific roles of *CCNE1* and *CCNE2* in regulating meiosis and fertility control, we validated their functions using RNA interference (RNAi) and overexpression techniques. This work establishes a theoretical foundation for developing novel rainbow trout strains exhibiting sterility or delayed maturation. Ultimately, by verifying the core functions of CCNE1 and CCNE2 in meiotic regulation and fertility modulation, we aim to provide a new theoretical basis for innovative rainbow trout breeding strategies.

## 2. Materials and Methods

### 2.1. Experimental Fish and Sample Collection

Healthy adult diploid rainbow trout (13-, 21-, and 35-month-old, *n* = 3) were obtained from the Bohai Experimental Station of the Heilongjiang River Fisheries Research Institute (Mudanjiang, China). The sizes of fish samples for each age group were as follows: 13-month-old (98.68 ± 18.68, 19.71 ± 1.86), 21-month-old (610.68 ± 38.41, 35.71 ± 3.26), and 35-month-old (2124.09 ± 118.41, 54.70 ± 4.29). Fish were transferred to 1 m^3^ recirculating aquaculture systems with conditions of 12 ± 0.2 °C, pH 7.0–7.3, and dissolved oxygen 7.8–10.0 mg/L, and fed twice daily. Fourteen tissues (the ovary, testis, fin ray, intestine, skin, heart, muscle, liver, eye, spleen, kidney, stomach, brain, and gill) were aseptically collected from euthanized specimens. Gonads were sampled at three developmental stages: 13 months (Stage II), 21 months (Stage III), and 35 months (Stage IV) [19]. All tissues were snap-frozen in liquid nitrogen and stored at −80 °C. Gonads from each age group were additionally fixed in 4% paraformaldehyde (PFA) for immunohistochemistry. RTG-2 cells were cultured at 18 °C under 5% CO_2_. All procedures complied with the European Union Directive (2010/63/EU) and followed the animal husbandry guidelines of Heilongjiang Province, China. Euthanasia was performed using MS-222 (100 mg/L), with all efforts made to minimize distress.

### 2.2. Molecular Cloning of CCNE

Gene-specific primers for cloning *CCNE1* and *CCNE2* were designed (Table 1). Following euthanasia with MS-222 (100 mg/L), ovarian tissue was aseptically collected from randomly selected rainbow trout. Total RNA was extracted using the TRIzol reagent and reverse-transcribed into cDNA with oligo (dT) primers. The cDNA served as a template for PCR amplification under the following conditions: initial denaturation at 94 °C for 2 min, 30 cycles of denaturation at 94 °C for 30 s, annealing at 61 °C (*CCNE1*) or 64 °C (*CCNE2*) for 30 s, and extension at 72 °C for 30 s, followed by a final extension at 72 °C for 5 min. Amplified products were ligated into pMD^TM^-18T vectors (Takara Bio, Beijing, China) and verified by Sanger sequencing.

### 2.3. Bioinformatics Analysis of CCNE

We performed multiple sequence alignment and open reading frame (ORF) identification using BLAST (v2.13.0) (https://blast.ncbi.nlm.nih.gov. visit on 20 February 2025). Structural features were analyzed using SignalP-5.0 for signal peptides (https://services.healthtech.dtu.dk/service.php?SignalP-5.0. visit on 20 February 2025), and predicted transmembrane helices with analyzed using TMHMM-2.0 (https://services.healthtech.dtu.dk/service.php?TMHMM-2.0. visit on 20 February 2025). Protein physicochemical properties (including molecular weight and isoelectric point) were determined using ExPASy ProtParam (https://web.expasy.org/protparam. visit on 20 February 2025). Tertiary structures were predicted with AlphaFold2, while sequence alignments were conducted in BioEdit (v7.2.5). Finally, a phylogenetic tree was constructed in MEGA11, using the neighbor-joining method with 1000 bootstrap replicates for topology validation.

### 2.4. Quantitative Real-Time PCR(q-PCR)

Total RNA was extracted from 14 rainbow trout tissues and fertilized eggs at various developmental stages, and gonads were taken from three healthy rainbow trout aged 13, 21, and 35 months. First-strand cDNA was synthesized using the PrimeScript^TM^ RT Master Mix (Takara, Dalian, China) following the manufacturer’s protocol, where the concentration was 1000 ng/μL. For each cDNA sample, quality evaluation was conducted using agarose gel electrophoresis. Quantitative PCR (qPCR) reactions were performed by CFX96 C1000 touch Thermal Cycler (Bio-RAD, Hercules, CA, USA), with 10 μL volumes containing 5 μL of 2 × S6 Universal SYBR qPCR Mix (Xinbei Bio, Shanghai, China), 0.4 μL of the primer mixture, and 1 μL of cDNA, using a CFX96 C1000 touch Thermal Cycler (BIO-RAD, USA). The primer sequences are provided in Table 2, and β-actin served as the internal reference gene. The thermal cycling protocol comprised initial denaturation at 95 °C for 30 s, followed by 40–45 cycles of denaturation (95 °C for 3–10 s) and annealing/extension (60 °C for 10–30 s). Experiments included three biological replicates for accuracy, and data were analyzed using the 2^−ΔΔCt^ method.

### 2.5. Immunohistochemistry

Gonads at different developmental stages were fixed in 4% paraformaldehyde for 24 h at 4 °C, embedded in paraplast, and sectioned at a thickness of 5 μm. Sections were deparaffinized in xylene, rehydrated through a graded ethanol series (100% to 70%), and underwent antigen retrieval in EDTA(C_10_H_16_N_2_O_8_) buffer (pH 8.0) at 95 °C for 20 min. Endogenous peroxidase activity was blocked with 3% H_2_O_2_ (10 min, room temperature), followed by incubation with 3% bovine serum albumin (BSA) in PBS (1×, 1 h, 37 °C) to prevent non-specific binding. Tissue sections were then incubated overnight at 4 °C for 16 h with primary antibodies against CCNE1/CCNE2 (anti-CCNE1/CCNE2, 1:200 dilution, ABclonal, Wuhan, China) and subsequently with HRP-conjugated secondary antibodies (1:1000 dilution, ABclonal) for 1 h at 37 °C. Target proteins were visualized using diaminobenzidine (DAB) chromogen (Servicebio, Wuhan, China) for 5 min, followed by hematoxylin counterstaining (30 s) for nuclear labeling. Finally, sections were dehydrated through an ascending ethanol series (70–100%), cleared in xylene, and mounted with neutral balsam for microscopic analysis (Nikon E100, Shanghai, China).

### 2.6. RNA Interference and Overexpression of CCNE

siRNAs targeting CCNE and its overexpression vector (pcDNA3.1) were designed and synthesized by Sangon Biotech (Shanghai, China). The siRNA sequences are provided in Table 1. For transfection, plasmid DNA and the Advanced^TM^ DNA/RNA Transfection Reagent (ZETA-life, Beijing, China) were mixed in a 1:1 ratio and introduced into RTG2 cells. mRNA expression following knockdown and overexpression was quantified using real-time PCR.

Protein expression was analyzed by Western blotting. Proteins were extracted from lysed cells (1 × 10^7^ cells/300 μL), and the supernatant was fractionated. The sample and the BCA working solution were mixed in a 1:8 ratio and measured at OD570 nm, and the concentration of RIPA was adjusted to 1 mg/mL. After SDS-PAGE separation and membrane transfer, blots were blocked with 3% BSA in TBST, incubated with primary and HRP-conjugated secondary antibodies, and visualized using chemiluminescent detection.

### 2.7. Determination of RTG2 Viability

We detected changes in RTG2 cell viability before and after *CCNE* knockdown and overexpression using a CCK-8 kit (Biosharp, Shanghai, China). We set up the experiment with 9 groups containing 5 replicate wells each: the blank group (MEM and CCK-8 solution), the 0 dosing group (RTG2, MEM, and CCK-8 solution), 6 dosing groups (RTG2-BLANK, siRNA-NC, siRNA-*CCNE1*, siRNA-*CCNE2*, pcDNA3.1(+), pcDNA3.1-*CCNE1*, MEM, and CCK-8 solution), and the anti-jamming group (CCK-8 solution). We spread RTG2 cells evenly into 96-well plates, and the hematocrit plate was adjusted to 2000 cells/well (100 μL/well) with conditions of 18 °C, 5% CO_2_ overnight culture, and 10 μ L of CCK-8 solution per well, and then incubated for 0, 4, 12, 24, 48, and 72 h. We measured the absorbance at 450 nm using a microplate reader (Molecular Devices, San Jose, CA, USA).

### 2.8. Immunofluorescence

RTG-2 cells were seeded in 24-well plates and transfected with CCNE-targeting siRNA or overexpression plasmids. After 24–48 h, cells were fixed with 4% paraformaldehyde (PFA), permeabilized with 0.5% Triton X-100 (ice bath, 10 min), and blocked with 5% fetal bovine serum (FBS) in 0.5% Triton X-100 (1 h, RT). Samples were then incubated with primary antibodies (4 °C, overnight) followed by fluorophore-conjugated secondary antibodies (RT, 1 h). Nuclei were counterstained with DAPI (5 μg/mL, 10 min), and images were acquired using fluorescence microscopy (YODN Hyper E500, OLYMPUS, Tokyo, Japan).

### 2.9. EDU Proliferation Test

Cell proliferation following CCNE knockdown or overexpression was assessed using the BeyoClick™ EdU-555 Kit (Beyotime, Shanghai, China). Transfected RTG-2 cells were seeded in 24-well plates and incubated with 2× EdU working solution (10 μM final concentration, 24 h). Cells were then fixed with 4% PFA, permeabilized with 0.5% Triton X-100 (ice bath, 10 min), and incubated with a Click reaction cocktail (86 μL Click Reaction Buffer, 4 μL CuSO_4_, 0.2 μL Azide 555, and 10 μL Click Additive Solution; RT, 30 min, protected from light). Nuclei were stained with Hoechst 33342 (5 μg/mL, 15 min), and proliferation was quantified via fluorescence microscopy (YODN Hyper E500, OLYMPUS, Japan).

### 2.10. Meiosis and Reproduction-Related Gene Testing

We detected changes in the expression of meiosis-related genes and reproduction-related genes after *CCNE* knockdown and overexpression using real-time PCR, as shown in Table 2 for key meiotic and reproduction-related primer information.

### 2.11. Statistical Analysis

Experiments were performed with three biological replicates. Data are presented as the mean ± standard deviation (SD) and visualized as histograms using GraphPad Prism 8.0.2. Statistical significance was determined by one-way ANOVA in IBM SPSS Statistics 25 (SPSS Inc., Chicago, IL, USA), using qPCR results to determine statistical significance. For Western blot analysis, the X-ray films were scanned, and band intensities were quantified as integrated optical density (IOD) values using ImageJ software 1.8.0. Statistical significance was defined as * *p* < 0.05, ** *p* < 0.01, and *** *p* < 0.001.

## 3. Results

### 3.1. Cloning and Sequence Analysis of the CCNE1 and CCNE2

**Figure 1:**
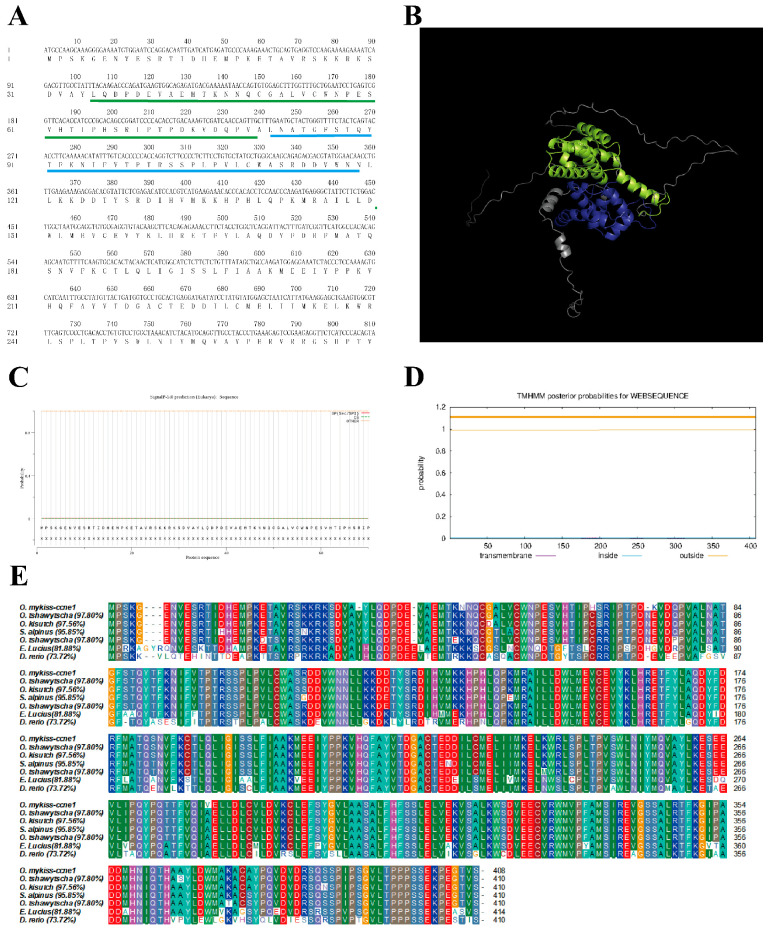
Bioinformatics analysis of *CCNE1*. (**A**). Nucleotide and amino acid sequences (the underlined regions are the two “cyclin boxes”, measuring 103–239 bp and 243–356 bp). The highlighted areas represent the specific marker sequences of cyclins, namely “cyclin box”, which are 103–239 bp (green) and 243–356 bp (blue). (**B**). Signal peptide prediction. (**C**). Transmembrane structural domain prediction. (**D**). Protein tertiary structure prediction. (**E**). Amino acid sequence homology analysis.

In this study, we cloned *CCNE1* and *CCNE2* from the ovarian tissue of rainbow trout, with ORFs of 1230 bp (GenBank PV25875) and 1188 bp (GenBank PV25199). We calculated the molecular formulas of *CCNE1* and *CCNE2* as C_2088_H_3249_N_537_O_610_S_26_ and C_2050_H_3217_N_549_O_588_S_21_, respectively. ExpASy analysis of the physicochemical properties of CCNE1 proteins showed a molecular weight of 46.4 kD, a pI of 5.53, a grand mean of −0.181 for hydrophilicity, and an instability coefficient of 58.43. The other isoform, CCNE2, had a molecular weight of 45.6 kD, a pI of 7.15, a grand mean of −0.358 for hydrophilicity, and an instability coefficient of 56.57, indicating that CCNE1 and CCNE2 are hydrophilic proteins and are unstable. As shown in Figure 1A and Figure S1A, the underlined cyclin box domain represents the signature motif of cell cycle regulators. No signal peptides were predicted, and both proteins were classified as non-transmembrane proteins. The tertiary structures (Figure 1D and Figure S1D) revealed predominant α-helices and random coils, with two conserved cyclin fold domains (green and blue highlights).

**Figure 2:**
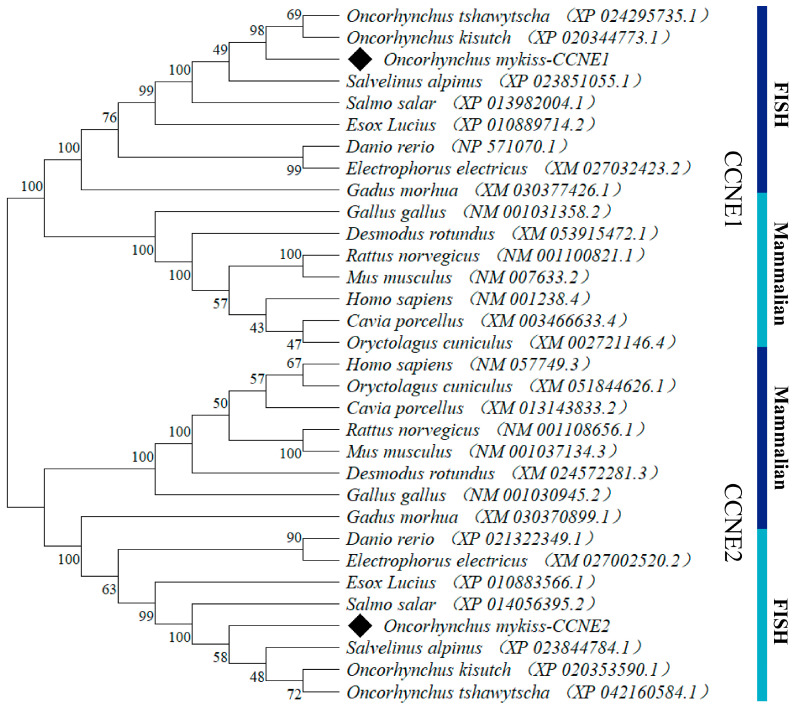
Phylogenetic tree of rainbow trout *CCNE* and the *CCNE* of other organisms based on their amino acid sequences. The diamond indicated CCNE1 and CCNE2 of rainbow trout.

The phylogenetic analysis (Figure 1E and Figure S1E) demonstrated >95% amino acid identity with other salmonids, indicating strong evolutionary conservation. *CCNE1* showed the highest homology with *Oncorhynchus tshawytscha* (97.80%) and the lowest with *Danio rerio* (73.72%), while *CNNE2* exhibited maximal identity with *Oncorhynchus kisutch* (98.48%) and minimal identity with D. rerio (75.19%). The phylogenetic analysis of the amino acid sequences of CCNE with other bony fish and mammalian homologs is shown in Figure 2. It shows that in bony fish, CCNE1 and CCNE2 are clustered with mammalian CCNE1 and CCNE2, respectively.

### 3.2. Characterization of CCNE1 and CCNE2 Expression in Different Tissues and at Different Times of Gonadal Development

**Figure 3:**
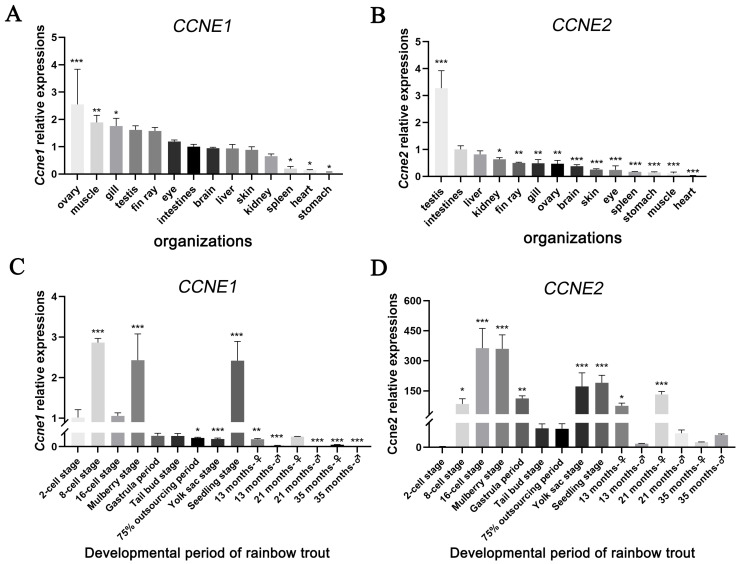
Expression of *CCNE1* and *CCNE2* in various tissues of rainbow trout during the period of fertilized egg development and the critical period of gonadal development. (**A**,**B**). Expression of CCNE1 and CCNE2 in various tissues of rainbow trout. (**C**,**D**). Expression of CCNE1 and CCNE2 in various tissues of rainbow trout during the period of fertilized egg development and the critical period of gonadal development. * *p* < 0.05, ** *p* < 0.01, *** *p* < 0.001.

The expression characteristics of *CCNE1* and *CCNE2* for different tissues of rainbow trout are shown in Figure 3. *CCNE1* showed the highest expression in the ovary with a value of 2.55, and lowest (with a value of 0.06) in the stomach, with ovarian expression being 42-fold higher than in the stomach (Figure 3A). *CCNE2* showed peak expression in the testis with a value of 3.28 and minimal expression in the heart (with a value of 0.03), demonstrating a 109-fold higher expression in the testis than in the heart (Figure 3B).

Developmental expression patterns are shown in Figure 3C,D. *CCNE1* expression peaked at the eight-cell stage with a value of 2.86 and reached its nadir in 21-month-old male gonads. During critical gonadal development periods, expression levels were consistently higher in females than males. *CCNE2* displayed maximal expression at the morula stage with a value of 363.9 and minimal expression at the two-cell stage with a value of 1.02. At 13 and 21 months, female expression exceeded male levels, while at 35 months, this pattern reversed.

### 3.3. IHC Staining of the Localization of CCNE1 and CCNE2 Proteins in the Gonad

**Figure 4:**
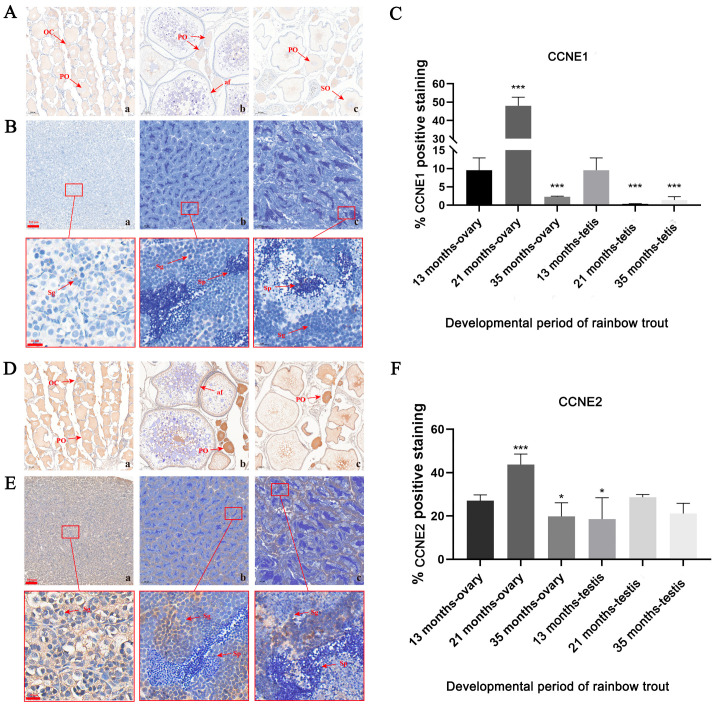
Distribution of CCNE1 (**A**,**B**) and CCNE2 (**D**,**E**) protein expression in rainbow trout gonads at different developmental periods. (**A**,**D**) Rainbow trout ovary tissue. (**B**,**E**) Rainbow trout spermatogonial tissue. (**a**) 13 months of age. (**b**) 21 months of age. (**c**) 35 months of age. (**C**,**F**) Positive rate of CCNE1 and CCNE2 proteins in rainbow trout gonadal tissues. OC: oocyte. PO: primary oocyte. SO: secondary oocytes. af: atretic follicles. Sg: spermatogonia. Sp: spermatozoa. Scale bars 200 µm, 20 µm. * *p* < 0.05, *** *p* < 0.001.

Figure 4A,B demonstrate CCNE1 protein expression in rainbow trout gonads across developmental stages (13, 21, and 35 months). At 13 months, strong nuclear and cytoplasmic immunostaining (brown-yellow) appeared in oogonia during primary oocyte differentiation (Figure 4A(a)). By 21 months, the signal intensity increased predominantly in primary oocyte nuclei with emerging atretic follicles (Figure 4A(b)). At 35 months, nuclear-localized signals diminished as secondary oocytes developed and signals became concentrated in the cytoplasm (Figure 4A(c)). In testicular tissues, peak immunoreactivity occurred in spermatogonia at 13 months, with signals concentrated in the cytoplasm of a small number of cells (Figure 4B(a)), and significant signal reduction was observed at 21 and 35 months (Figure 4B(b,c)).

As shown in Figure 4D,E, CCNE2 protein expression in rainbow trout ovarian tissues at 13, 21, and 35 months of age showed signal intensities comparable to CCNE1. Expression peaked at 21 months, with the main signal concentrated in primary oocytes. Lower levels were seen at 13 and 35 months, with expression observed in both the cytoplasm and nucleus (Figure 4D). In testicular tissues, CCNE2 exhibited significantly weaker immunoreactivity than in the ovaries, reaching minimal levels at 13 months, with expression visible in both the cytoplasm and nucleus, peaking at 21 months with signals concentrated in spermatogonial cells and declining by 35 months (Figure 4E).

### 3.4. Validation of CCNE1 and CCNE2 siRNA with Overexpression

**Figure 5:**
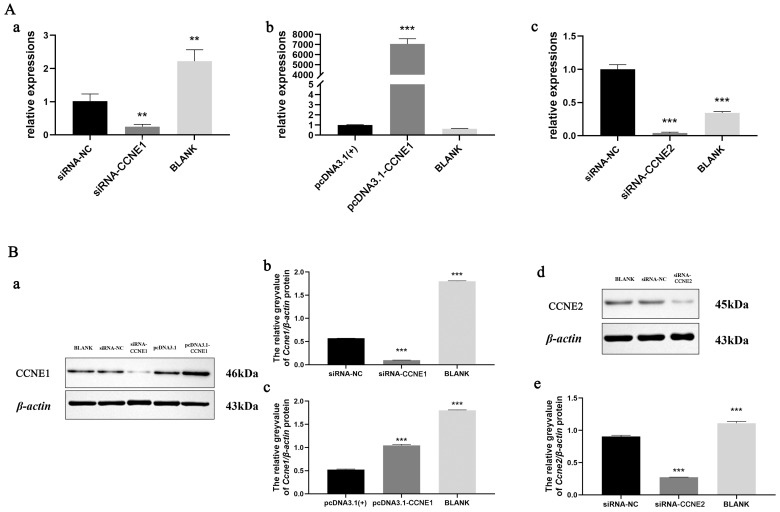
Validation of *CCNE* siRNA with the overexpression vector. (**A**). Real-time fluorescence quantification. (**a**) RTG2 expression after *CCNE1* knockdown. (**b**) RTG2 expression after overexpression of *CCNE1*. (**c**) RTG2 expression after *CCNE2* knockdown. (**B**). Western blot. (**a**,**d**): Protein expression WB bands. (**b**,**c**,**e**): *CCNE1, CCNE2*/*β-actin* relative grey values. ** *p* < 0.01, *** *p* < 0.001.

Quantitative PCR analysis of *CCNE1* and *CCNE2* expression in RTG-2 cells following knockdown or overexpression revealed significant changes. Results showed that the siRNA-*CCNE1* group exhibited significantly lower expression than both the siRNA-NC and blank groups. The control group is 9.09 times higher than the knockout group (Figure 5A(a)). The pcDNA3.1-*CCNE1* overexpression group showed markedly higher expression than the pcDNA3.1 (+) empty vector and blank groups (*p* < 0.001, Figure 5A(b)). Similarly, the siRNA-*CCNE2* group had significantly reduced expression versus controls, and the blank group is 24.3 times higher than the knockdown group (Figure 5A(c)).

Western blot analysis of CCNE1 and CCNE2 protein expression in RTG-2 cells after interventions yielded consistent results (Figure 5B(a)). Results showed that *CCNE1* knockdown significantly reduced protein expression. Compared to the knockdown group, the control group showed 5.73-fold higher expression (Figure 5B(b)). CCNE1 overexpression substantially increased protein levels. Relative to the overexpression group, the empty vector group showed 1.99-fold lower expression (Figure 5B(c)). CCNE2 knockdown resulted in markedly decreased protein expression versus control groups (Figure 5B(e)).

### 3.5. Cell Viability Assay

We assessed RTG-2 cell viability using the CCK-8 assay at 0, 4, 12, 24, 48, and 72 h post-knockdown or post-overexpression. As shown in Figure S2, viability increased over time in all groups. The *CCNE1* and *CCNE2* knockdown groups exhibited the lowest viability, followed by the control and empty vector groups, which showed comparable viability. The *CCNE1* overexpression group demonstrated the highest viability throughout the experiment. At 72 h, viability in the *CCNE1* overexpression group was 2-fold higher than both the control and empty vector groups (*p* < 0.01). The control group showed 1.7-fold higher viability than the *CCNE1* and *CCNE2* knockdown groups (*p* < 0.01).

### 3.6. Localization of CCNE1 and CCNE2 Proteins in RGT2

**Figure 6:**
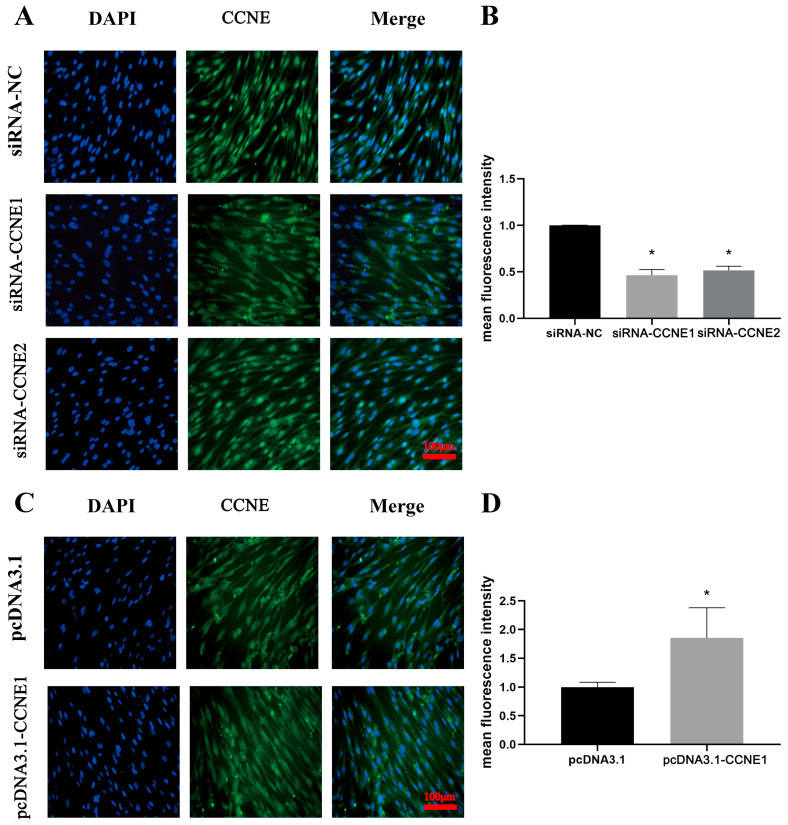
Protein expression of RTG2 cells after *CCNE1* and *CCNE2* knockdown and overexpression. (**A**). Protein localization of RTG2 after *CCNE1* and *CCNE2* knockdown (green fluorescence). (**B**). Average fluorescence intensity after *CCNE1* and *CCNE2* knockdown (* *p* < 0.05). (**C**). Protein localization of RTG2 after *CCNE1* and *CCNE2* overexpression (green fluorescence). (**D**). Average fluorescence intensity after *CCNE1* overexpression (* *p* < 0.05). Blue fluorescence represents the cell nucleus of the cell, while green fluorescence indicates the expression of the gene within the cell.

Immunofluorescence was performed to assess CCNE1 and CCNE2 protein expression in RTG-2 cells following knockdown or overexpression (Figure 6). Both proteins were localized predominantly to the nucleus. The results of the five groups were analyzed, and it was found that the average fluorescence intensity of cells with CCNE1 and CCNE2 knockdown was significantly lower than that of the control group. The average fluorescence intensity of the control group was 2.1 and 1.9 times higher than that of the CCNE1 and CCNE2 knockdown groups. The average fluorescence intensity of cells in the CCNE1 overexpression group was significantly higher than that in the control group, and the average fluorescence intensity in the empty load group was 1.8 times that of the CCNE1 overexpression group.

### 3.7. Cell Proliferation Assay

**Figure 7:**
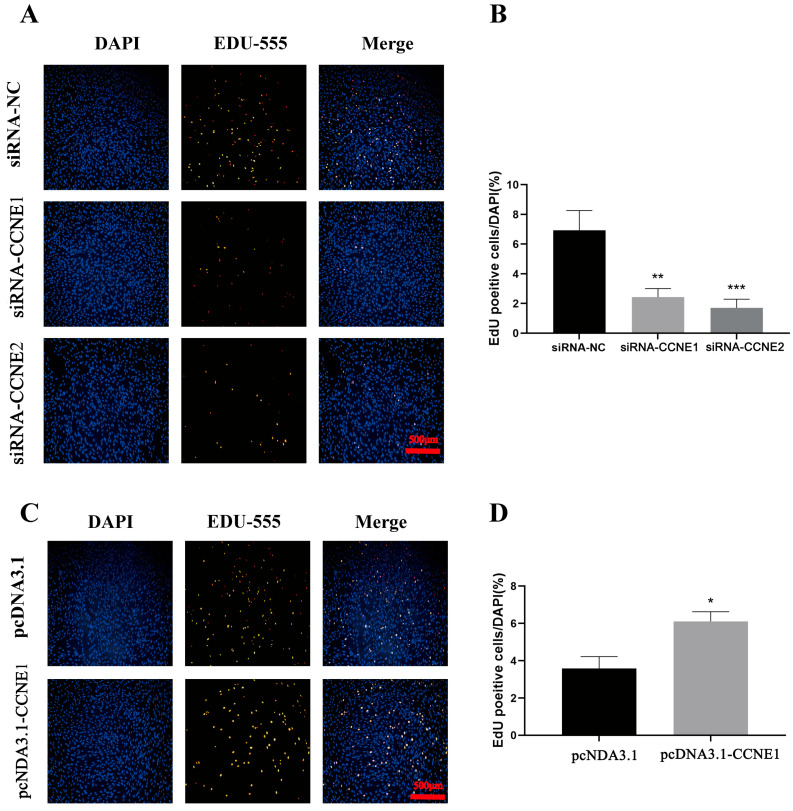
Cell proliferation expression of RTG2 after *CCNE1* and *CCNE2* knockdown and overexpression. (**A**). Cell proliferation of RTG2 after *CCNE1* and *CCNE2* knockdown (red fluorescence). (**B**). Number of positive cells/DAPI (%) after *CCNE1* and *CCNE2* knockdown (** *p* < 0.01, *** *p* < 0.001). (**C**). *CCNE1*, cell proliferation of RTG2 after *CCNE2* overexpression (red fluorescence). (**D**). Number of positive cells/DAPI (%) after *CCNE1* overexpression (* *p* < 0.05). Blue fluorescence represents the cell nucleus of the cell, while green fluorescence indicates the expression of the gene within the cell.

We assessed proliferative activity in RTG-2 cells using EdU assays following CCNE1 and CCNE2 knockdown or overexpression. As shown in Figure 7, proliferating cells (EdU-positive, red fluorescence) exhibited significant changes. *CCNE1* and *CCNE2* knockdowns showed significantly reduced EdU-positive cell counts versus controls. CCNE1-overexpressing cells displayed markedly increased EdU-positive cells compared to controls.

### 3.8. Expression of Reproductive and Meiotic Genes

**Figure 8:**
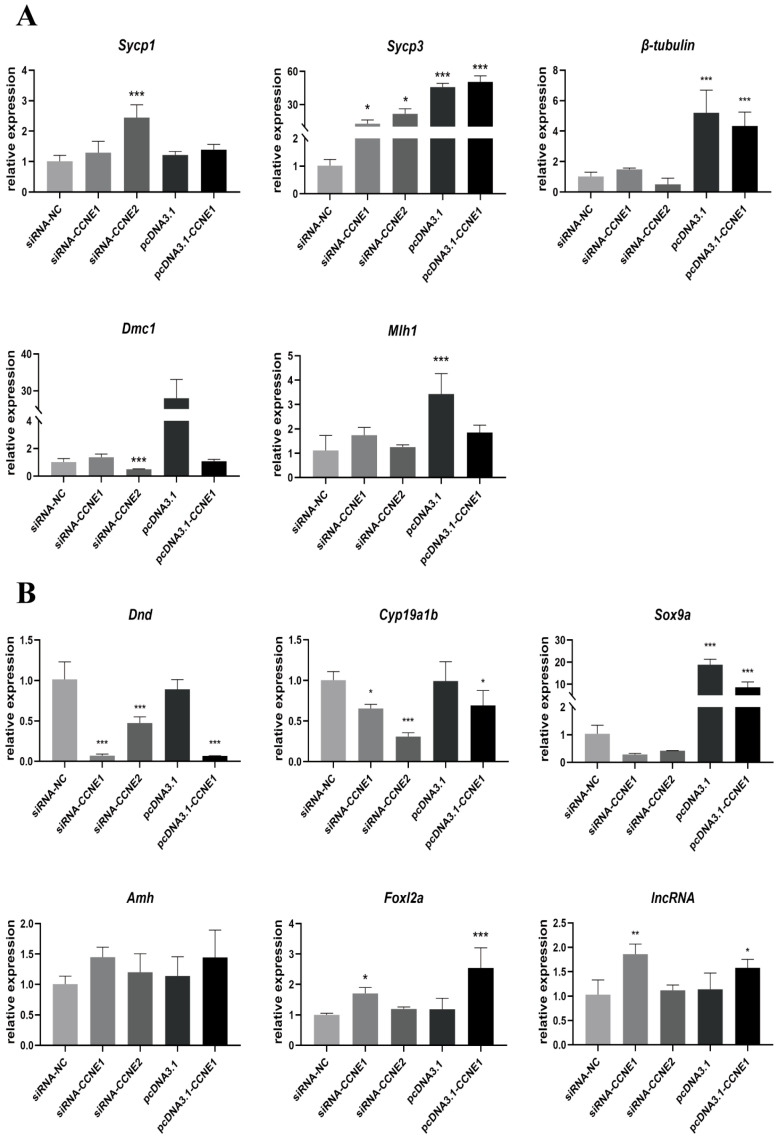
Expression of key meiotic and reproduction-related genes upon *CCNE1* and *CCNE2* knockdown and overexpression (* *p* < 0.05, ** *p* < 0.01, *** *p* < 0.001). (**A**). Key meiotic genes. (**B**). Reproduction-related genes.

Figure 8A shows RTG2 expression changes in five meiosis-related genes after *CCNE1* and *CCNE2* knockdown and overexpression. *sycp1* and *sycp3* expression increased after *CCNE1* and *CCNE2* knockdown, and *CCNE1* knockdown upregulated *β-tubulin*, *Dmc1,* and *M1h1* expression. In contrast, *CCNE1* overexpression or *CCNE2* knockdown inhibited its expression, antagonizing *CCNE1* and acting synergistically with *CCNE2.*

Figure 8B illustrates RTG2 expression changes in six reproduction-related genes after *CCNE1* and *CCNE2* knockdown/overexpression. The expression of three genes, *dnd*, *cyp19a1b*, and *sox9a*, were all downregulated. Expression of *amh*, *fox12a*, and *lncRNA-MSTRG.74687* was not affected by *CCNE2* knockdown, and their expression increased with *CCNE1* overexpression.

## 4. Discussion

### 4.1. Sequence Analysis of CCNE1 and CCNE2 in Rainbow Trout

The Cyclin E protein exists in most species and functions as a nuclear regulator that binds cdk2 to control the transcription of cell cycle-related genes. Two isoforms exist with high structural similarity but divergent functional domains, suggesting potential isoform-specific roles [20]. The cyclin box, consisting of the C-terminal of Cyclin C and the N-terminal of Cyclin N, is the core conserved structure of the cell cycle protein that is used to specifically recognize the cell cycle kinase molecule [21]. The CCNE1 and CCNE2 genes share 47% amino acid identity overall and 70% sequence identity within their cyclin box domains [22], reflecting conserved regulatory functions. The open reading frames (ORFs) of *CCNE1* and *CCNE2* are 1230 bp and 1188 bp, encoding 408 and 395 amino acids, respectively, and contain two cyclin boxes that are required for CDK binding. Predicted tertiary structures show high similarity (>95% α-helix/coil composition). The amino acid sequences were all >95% identity compared to other salmonids, indicating that both *CCNE1* and *CCNE2* have evolved with significant amino acid conservation. Phylogenetic analysis clusters rainbow trout *CCNE1*/*CCNE2* with *O. tshawytscha* and *O. kisutch*. Mammalian cyclins form a distinct clade, consistent with vertebrate evolutionary relationships.

### 4.2. Expression of CCNE1 and CCNE2 in Rainbow Trout

Although *CCNE1* and *CCNE2* were expressed in all tissues, their levels increased significantly in the ovary and testis, suggesting specialized functions in these organs, consistent with reports in *P. monodon* where ovarian enrichment suggests critical roles in oogenesis and ovarian development [14]. Conversely, *CCNE2* showed the highest expression in the testis (*p* < 0.001), and both *CCNE* genes were found to be expressed in spermatogonia, where the protein content of *CCNE2* was higher than that of *CCNE1*, as shown by previous authors [15], indicating potential specialization in spermatogenic regulation.

Developmental profiling in rainbow trout revealed the ubiquitous expression of both *CCNE1* and *CCNE2* throughout fertilized egg maturation and gonadal development, indicating their functional involvement across all reproductive stages. CCNE1 protein expression peaked at the eight-cell stage (early embryogenesis), coinciding with rapid mitotic divisions in compact blastomeres [23] and suggesting participation in cell cycle regulation. *CCNE1* expression showed a trend of increasing and then decreasing in the ovary. Ovarian transcript levels followed an unimodal pattern, peaking at 21 months (Stage III vitellogenesis) when nutrient accumulation accelerates. This aligns with peak *CCNE1* expression during Stage III ovarian development in Exopalaemon carinicauda [13], implicating conserved functions in oocyte maturation and nutrient transport. Testicular expression declined after 13 months (the spermatogonia II stage), coinciding with gonadal differentiation completion [24] and indicating potential roles in early spermatogenesis. *CCNE2* abundance peaked at the morula stage, characterized by enhanced cell adhesion and compaction [23], which is consistent with functions in cell division coordination. Testicular expression peaked at 21 months (Stage III) during primary spermatocyte proliferation, consistent with its established role in chromatin replication complex assembly and mitotic control [25,26].

### 4.3. Immunohistochemical Analysis of Protein Expression

In this paper, CCNE1 and CCNE2 proteins were detected at 13, 21, and 35 months, corresponding to gonadal stages II, III, and IV, respectively. CCNE1 was localized in the cytoplasm and nucleus in the ovary, suggesting that it may be involved in the initiation of DNA replication and cellular differentiation. Signals were seen in the oogonia, primary oocytes, and secondary oocytes, and the cytoplasmic signals weakened over time, revealing a shift in function from proliferation regulation to yolk substance synthesis and reserve. Protein positivity analyses revealed that CCNE1 peaks at the stage of vitellogenesis (Stage III), suggesting that it may drive oocyte growth and yolk deposition by regulating the G1/S phase transition and nutrient metabolism pathways such as yolk precursor protein synthesis. Consistent with Zhang’s findings in the ridge-tailed white shrimp, CCNE1 expression was highest at Stage III [13], revealing a critical role for CCNE1 in yolk production, and extremely weak signals in the spermatophore, suggesting that it plays a lesser role or even has no effect in spermatophore development. CCNE2 protein signals were significantly higher than CCNE1, and the protein expression trend in the ovary was similar to that of CCNE1, but with stronger signals, which may enhance the regulation of the oocyte cycle and yolk synthesis or compensate for some of the functions of CCNE1 by binding to cdk2 to form a complex. CCNE2 was specifically and highly expressed in spermatid III (the dominant stage of primary spermatocytes), which suggests that spermatogonial is linked to CCNE2 specifically, and is highly expressed in spermatogonia III (primary spermatocyte-dominant stage), promoting spermatogonia differentiation to primary spermatocytes and regulating meiosis initiation. Its expression is highly coincident with spermatogenesis, which may be directly involved in homologous chromosome separation or spermatogenesis. These results indicate that spermatogenesis operates primarily through CCNE2-dominated pathways, while oogenesis utilizes both CCNE1 and CCNE2, reflecting a fundamental divergence in gametogenic regulation.

### 4.4. Biological Functional of CCNE1 and CCNE2 at the Cellular Level

The rainbow trout ovary cell line (RTG2), established as the world’s first fish cell line, remains a premier model for in vitro studies of gonadal function [27]. Using a plasmid transfection reagent, siRNA and an overexpression plasmid were transfected into RTG2 cells, and mRNA and protein levels were examined after 72 h of culture. It was found that the expected results were achieved, with a significant decrease in RTG2 mRNA and protein expression in the knockdown group and a significant increase in RTG2 expression in the overexpression group, laying a foundation for subsequent genetic research at the cellular level.

Currently, RNAi and overexpression technologies have been widely used in the verification of gene function in aquatic animals. Liu et al. injected *CCNE1* dsRNA into rainbow trout, which significantly reduced the expression of *CCNE1* and suppressed the expression of key meiotic genes, revealing its central role in gonadal development [28]. In this study, we found that *CCNE1* and *CCNE2* were positively correlated with RTG2 cell viability. RGT2 cell viability decreased in the *CCNE1* and *CCNE2* knockdown group and increased in the *CCNE1* overexpression group, similar to the research results of Xie et al. (2005) [29]. Immunofluorescence analysis confirmed the nuclear localization of both CCNE1 and CCNE2 proteins. EDU proliferation assays demonstrated that cell proliferation was significantly inhibited by *CCNE1* and *CCNE2* knockdown, and overexpression of *CCNE1* cell proliferation was significantly increased, with a positive correlation, indicating that *CCNE* genes have positive regulatory roles in cell cycle progression. These findings suggest that CCNE1 and CCNE2 jointly support reproductive capacity through the direct promotion of gonadal cell proliferation and maintenance of gonadal homeostasis. However, the specific mechanism of how CCNE affects reproductive capacity still needs to be further determined through in vivo experiments throughout the entire lifecycle.

### 4.5. Quantitative Analysis of Genes Related to Reproduction and Meiosis

Meiosis is a specialized form of cell division essential for gametogenesis, involving homologous chromosome association, crossover recombination, and segregation. The synaptonemal complex proteins *sycp1* and *sycp3*, expressed only in meiosis, mediate homologous chromosome pairing and synapsis. During diplotene, *sycp1* diminishes at desynapsing chromosomal regions and fully dissociates thereafter, while *sycp3* persists on meiotic chromosomes until mid-metaphase I [30]. Notably, *Sycp1*-knockout mice exhibit sterility due to defective chromosome synapsis [31]. Following *CCNE1*/*CCNE2* knockdown, *sycp1* expression significantly increased, suggesting these cyclins suppress chromosome association through negative regulation. We propose that during leptotene-zygotene, *CCNE1*/*CCNE2* prevents premature synapsis by inhibiting *sycp*1/sycp3 expression. Conversely, their diminished activity post-diplotene may facilitate synaptonemal complex disassembly, consistent with reported *sycp*1 reduction during diplotene [31,32]. *β-tubulin* (β-microtubulin) is a conserved eukaryotic protein essential for meiotic spindle assembly [33]. *dmc1* (meiotic recombinase 1) is a meiosis-specific recombinase required for homologous chromosome association [34]. *m1h1* (MutL Homolog 1) is critical for crossover recombination and genome stability. Mlh1-deficient zebrafish display male sterility with meiotic arrest at metaphase I [35]. In this study, *CCNE1* knockdown upregulated *β-tubulin*, *dmc1*, and *mlh1* expression, whereas *CCNE*1 overexpression or *CCNE*2 knockdown suppressed them. This indicates that *CCNE1* acts as a negative regulator of core meiotic genes, potentially preventing aberrant meiotic initiation (e.g., prophase chromosome instability) by restraining premature gene expression. Conversely, *CCNE*2 appears to function synergistically with these genes. Its depletion caused spindle defects, impaired recombination, and gametogenesis arrest, phenocopying Mlh1-deficiency sterility [36]. Thus, *CCNE*2 likely maintains meiotic progression through positive regulation.

Reproductive development encompasses the entire process from germ cell maturation into functional gametes, critically influencing organisms through its regulation of gametogenesis, gonadal differentiation, and reproductive capacity. *dnd* is a specific germinal factor that is associated with the migration and movement of primordial germ cells (PGCs), and the destruction of PGCs affects the fertility of individuals [37]. *cyp19a1b* is one of the isoforms of aromatase that converts androgens to estrogens [38]. *sox9a* is a member of the sox superfamily and is associated with testis formation and differentiation [39]. Notably, dnd, cyp19a1b, and sox9a expression decreased following either *CCNE1* or *CCNE2* knockdown. This supports the hypothesis that *CCNE1* and *CCNE2* act as “reproductive regulatory hubs”, coordinating germ cell migration, proliferation, meiosis, and gamete maturation. Their deficiency may cause PGC insufficiency, aberrant gonadal differentiation, and estrogen imbalance, ultimately leading to reduced fertility or sterility. *amh* inhibits the development of Müllerian ducts in males, can inhibit the first meiosis, plays a role in male sex differentiation, and is involved in follicular development [40]. *fox12a* is an autosomal transcription factor maintaining ovarian function; its mutation causes premature ovarian failure and female sterility [39]. *lncRNA-MSTRG.74687* is involved in gene transcription and post-transcriptional regulation and plays a role in germ cell development and differentiation [18,41]. While *amh*, *foxl2a*, and lncRNA-*MSTRG.74687* expression remained unaffected by *CCNE2* knockdown, all three were upregulated upon *CCNE1* knockdown. This demonstrates *CCNE*’s pivotal role in maintaining gonadal function and gametogenesis, likely through the activation of sex differentiation and reproductive pathways.

These findings demonstrate that *CCNE1* and *CCNE2*, as key regulatory hubs in reproduction, orchestrate distinct molecular mechanisms to balance meiosis and germ cell development. Dysfunction of these genes may trigger reproductive disorders ranging from the defective migration of primordial germ cells to the blockage of gamete maturation. This study lays crucial groundwork for understanding infertility mechanisms and advancing reproductive regulation technologies.

## 5. Conclusions

In this study, two *Cyclin E* subtypes (*CCNE1* and *CCNE2*) were cloned and identified in rainbow trout. Expression and localization analyses revealed that *CCNE1* is predominantly and specifically expressed in ovarian tissue, while *CCNE2* shows predominant testicular-specific expression. The expression levels of both genes positively correlate with the viability and proliferation of rainbow trout gonadal cells (RTG-2). Both subtypes regulate gamete development by controlling key meiotic genes. Collectively, *CCNE1* and *CCNE2* participate in gametogenesis and reproductive regulation in rainbow trout, playing critical roles in meiosis and reproductive development. This functional characterization of *Cyclin E* provides a molecular framework for future analyses of fish reproductive regulation mechanisms. Future studies should validate these findings in in vivo models to elucidate the full physiological role of *CCNE1* and *CCNE2* in rainbow trout reproduction.

## Figures and Tables

**Table 1:** Sequence information of siRNA interference fragments of *CCNE*.

Groups	Sequence	Length/bp
siRNA-*Cyclin E1*	GGAACAACCUGUUGAAGAA	19
siRNA-*Cyclin E2*	GGAACAACCUGUUGAAGAA	19
NC	UUCUCCGAACGUGUCACGUTT	21

**Table 2:** Primer sequences used in this study.

Primer Name	Primer Sequence (5′-3′)	Usage
*CCNE1*-F	GCGGGAGAATATTTTTAGGGTCTAT	Partial mRNA cloning
*CCNE1*-R	TGCAATATCTTGGTCCTGTCTTGAG	
*CCNE2*-F	AGTGGATCATTTTCGGTGGAACTCT	
*CCNE2*-R	CAGAAGATCAACACGAGGAGACCCT	
*CCNE1*-qF	GTCTTCCCCTCTTCCTGTGCTA	Real-time PCR
*CCNE1*-qR	AGCCAGTCCAGAAGAATAGCCC	
*CCNE2*-qF	GTGGTCGCATCACATTGAAAGC	
*CCNE2*-qR	GACTTGCCCCTTCTTCTGACCA	
*β-actin-*F	CTCACCGACTACCTGATGAAGATC	
*β-actin-*R	GTAGCACAGCTTCTCCTTGATGTC	
*Amh*-F	GGGAATAACCATGCTATCCTGCTT	
*Amh* *-R*	CTCCACCACCTTGAGGTCCTCATAGT	
*Foxl2a* *-F*	TGTGCTGGATTTGTTTTTTGTT	
*Foxl2a*-R	GTGTCGTGGACCATCAGGGCCA	
*Cyp19a1b*-F	TGAGGAAGGCACTGGAAGATGAC	
*Cyp19a1b*-R	GGCTGGAAGAAACGACTGGGC	
*Dnd*-F	GCTAGGGAGAGAAAATAACTTGCAA	
*Dnd*-R	CTGTTTCTACATGCATCATTCCCAC	
*IncRNA*-F	AACGCCCAAACAAGGACT	
*IncRNA*-R	GCCACGAGGACATTGACA	
*Sycp1*-F	ACCGAAGCTCTCAGAACTCC	
*Sycp1*-R	TGTTCCGAGCTGTCAGACTT	
*Sycp3*-F	AGCCATGCAAGCCAAGAGAA	
*Sycp3*-R	GACAGTGGCCATCTCTTGCT	
*Mlh1*-F	TAAAGACCCAACCCAAACCC	
*Mlh1*-R	CTGCTCACCACCTCCACAAT	
*Dmc1*-F	CATCAGCAATTCCCCCTGGA	
*Dmc1*-R	GTTGGATTTTGTGGCAGCCA	
*β-tubulin*-F	TTGGATGTGGTGAGGAAAGA	
*β-tubulin*-R	ATAGGTGGGCGTGGTAAGTT	
*Sox9a*-F	CACATCTCTTCCGGTGACATC	
*Sox9a* *-R*	AAGTACTGGTCGAACTCATGGA	

## Data Availability

The original contributions presented in this study are included in the article/Supplementary Materials. Further inquiries can be directed to the corresponding authors.

## Supplementary Materials

The following supporting information can be downloaded at: https://www.mdpi.com/article/10.3390/biology14070862/s1. Figure S1: Bioinformatics analysis of CCNE2. Nucleotide and amino acid sequences (the underlined regions are the two “cyclin boxes” measuring 93–223 bp and 233–331 bp). B. Signal peptide prediction. C. Transmembrane structural domain prediction. D. Protein tertiary structure prediction. E. Amino acid sequence homology analysis. Figure S2: Changes in RTG2 cell viability after CCNE1 and CCNE2 knockdown and overexpression. The raw data supporting the conclusions of this article will be made available by the authors, without undue reservation.
