# Correction: Liu et al. Functional Analysis of the Cyclin E Gene in the Reproductive Development of Rainbow Trout (*Oncorhynchus mykiss*). *Biology* 2025, *14*, 862

**DOI:** 10.3390/biology15050408

**Published:** 2026-03-02

**Authors:** Enhui Liu, Haixia Song, Wei Gu, Gaochao Wang, Peng Fan, Kaibo Ge, Yunchao Sun, Datian Li, Gefeng Xu, Tianqing Huang

**Affiliations:** 1 Heilongjiang River Fisheries Research Institute, Chinese Academy of Fishery Sciences, Harbin 150070, China; liuenhui@hrfri.ac.cn (E.L.); songhx1998@163.com (H.S.); guwei@hrfri.ac.cn (W.G.); gaochaowang@ymail.com (G.W.); fanpeng@hrfri.ac.cn (P.F.); gekaibo@hrfri.ac.cn (K.G.); sunyunchao@hrfri.ac.cn (Y.S.); lidatian@hrfri.ac.cn (D.L.); 2 State Key Laboratory of Mariculture Biobreeding and Sustainable Goods, Harbin 150070, China; 3 College of Fisheries and Life Sciences, Dalian Ocean University, Dalian 116011, China

## Figure Legend

In the original publication [1], there was a mistake in the legend for Figure 4. AD should be identified as the “ovary” (not AB). BE should be identified as the “testis” (not DE). The correct legend appears below.

**Figure 4.** Distribution of CCNE1 (**A**,**B**) and CCNE2 (**D**,**E**) protein expression in rainbow trout gonads at different developmental periods. (**A**,**D**) Rainbow trout ovary tissue. (**B**,**E**) Rainbow trout spermatogonial tissue. (**a**) 13 months of age. (**b**) 21 months of age. (**c**) 35 months of age. (**C**,**F**) Positive rate of CCNE1 and CCNE2 proteins in rainbow trout gonadal tissues. OC: oocyte. PO: primary oocyte. SO: secondary oocytes. af: atretic follicles. Sg: spermatogonia. Sp: spermatozoa. Scale bars 200 µm, 20 µm. * *p* < 0.05, *** *p* < 0.001.

The authors state that the scientific conclusions are unaffected. This correction was approved by the Academic Editor. The original publication has also been updated.
