# Supplementary material for: Functional Analysis of the Cyclin E Gene in the Reproductive Development of Rainbow Trout (Oncorhynchus mykiss)
Source: Biology (Basel). 2025 Jul 16;14(7):862. doi: 10.3390/biology14070862 (PMC12292526; doi:10.3390/biology14070862)
Supplement: Supplementary file 1 [file biology-14-00862-s001.zip › biology-3715206-supplementary.pdf]

Supplementary Materials:

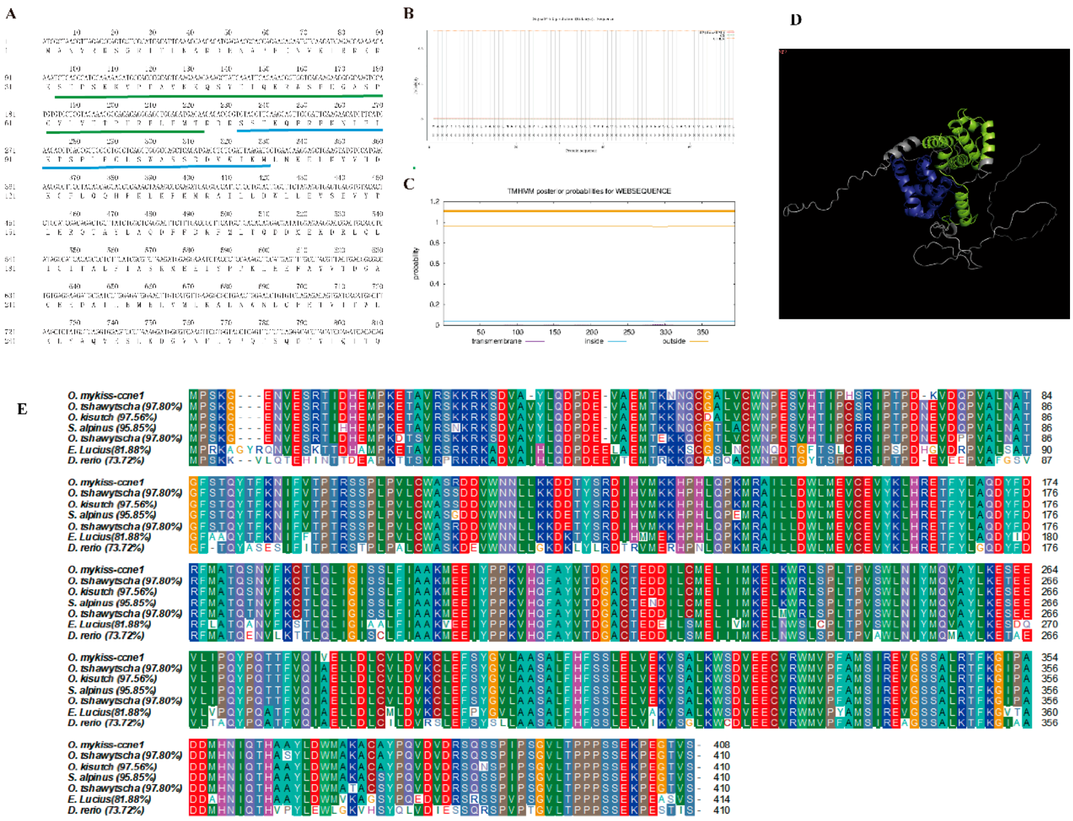

Figure S1. Bioinformatics analysis of the *ccne2*. A. Nucleotide and amino acid sequences (the underlined regions are the two ‘cyclin boxes’, 93-223 bp and 233-331 bp). B. Signal peptide prediction. C. Transmembrane structural domain prediction. D. Protein tertiary structure prediction. E. Amino acid sequence homology analysis.

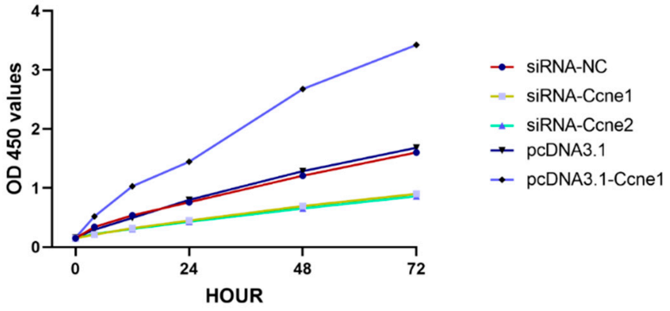

Figure S2. Changes in RTG2 cell viability after *ccne1* and *ccne2* knockdown and overexpression.
