# Supplementary figures and images for: Functional Analysis of the Cyclin E Gene in the Reproductive Development of Rainbow Trout (Oncorhynchus mykiss)
Source: Biology (Basel). 2025 Jul 16;14(7):862. doi: 10.3390/biology14070862 (PMC12292526; doi:10.3390/biology14070862)

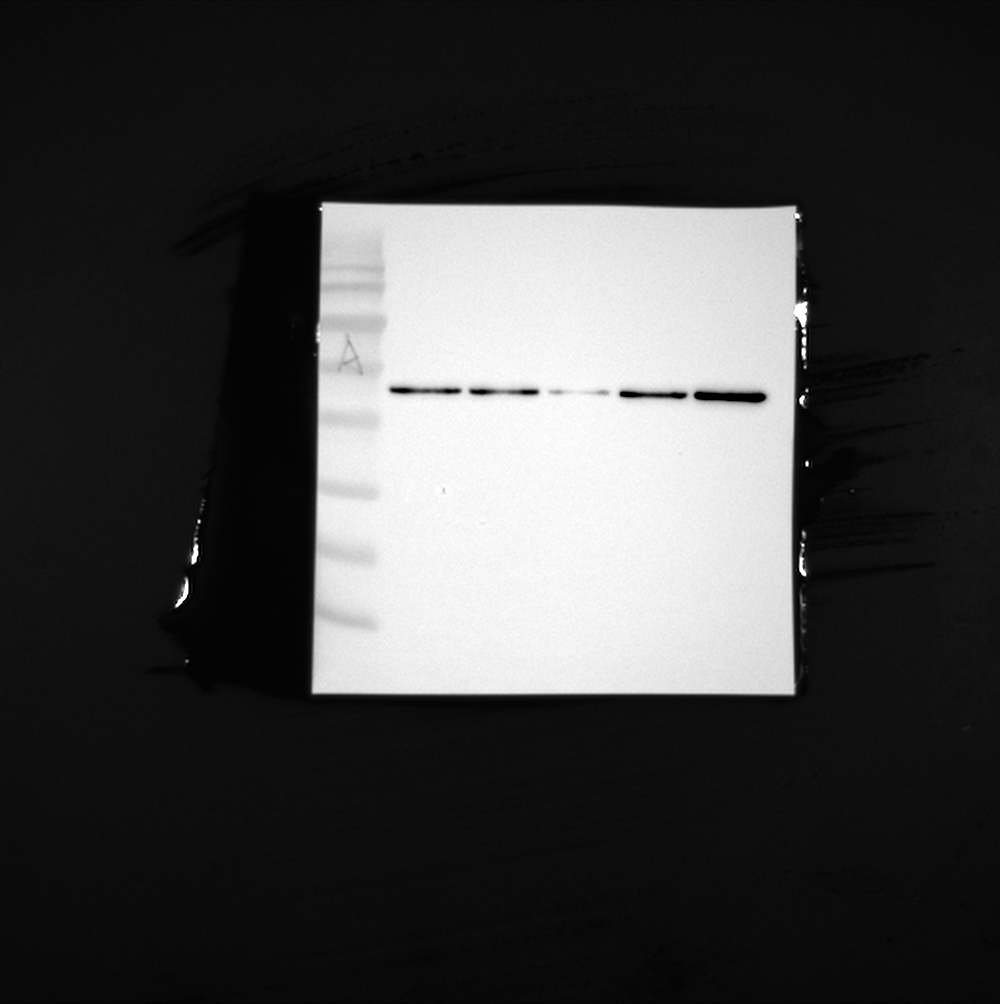

Supplement: Supplementary file 1 [file biology-14-00862-s001.zip › Cyclin E1.tif]

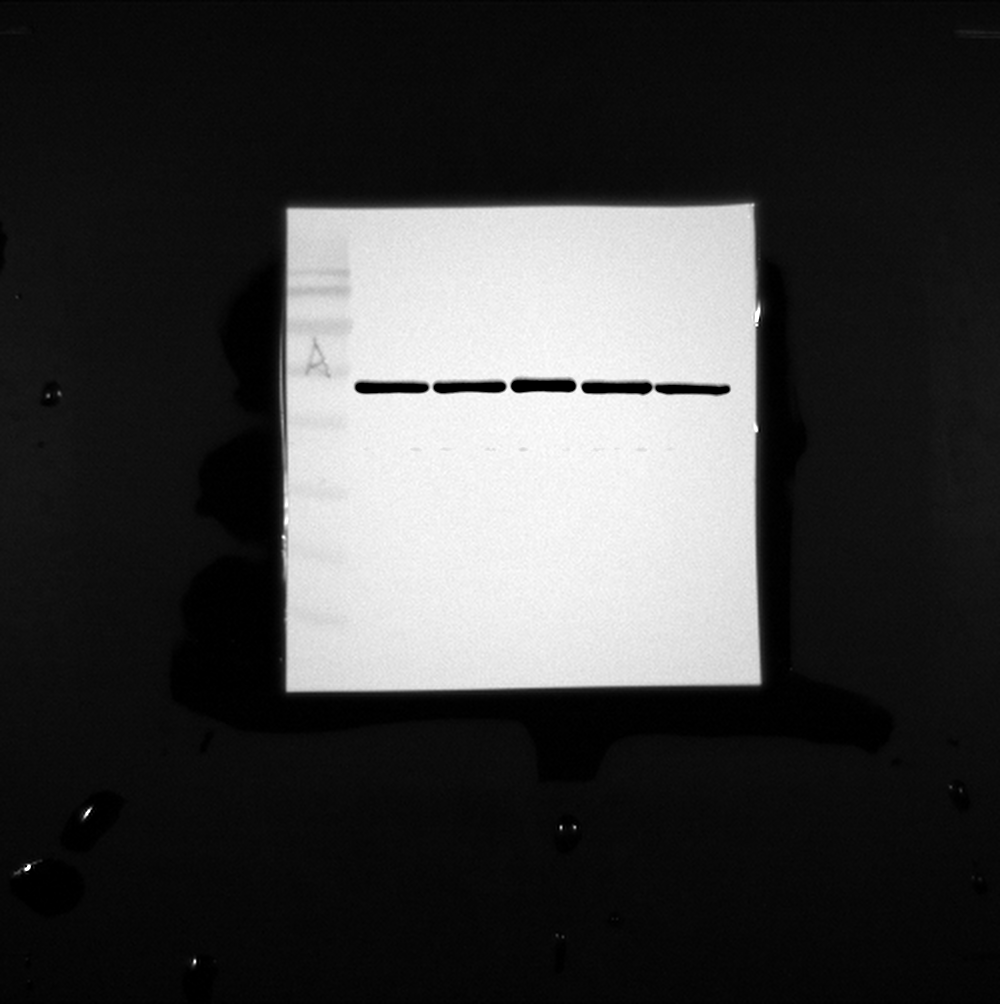

Supplement: Supplementary file 1 [file biology-14-00862-s001.zip › Cyclin E1β actin.tif]

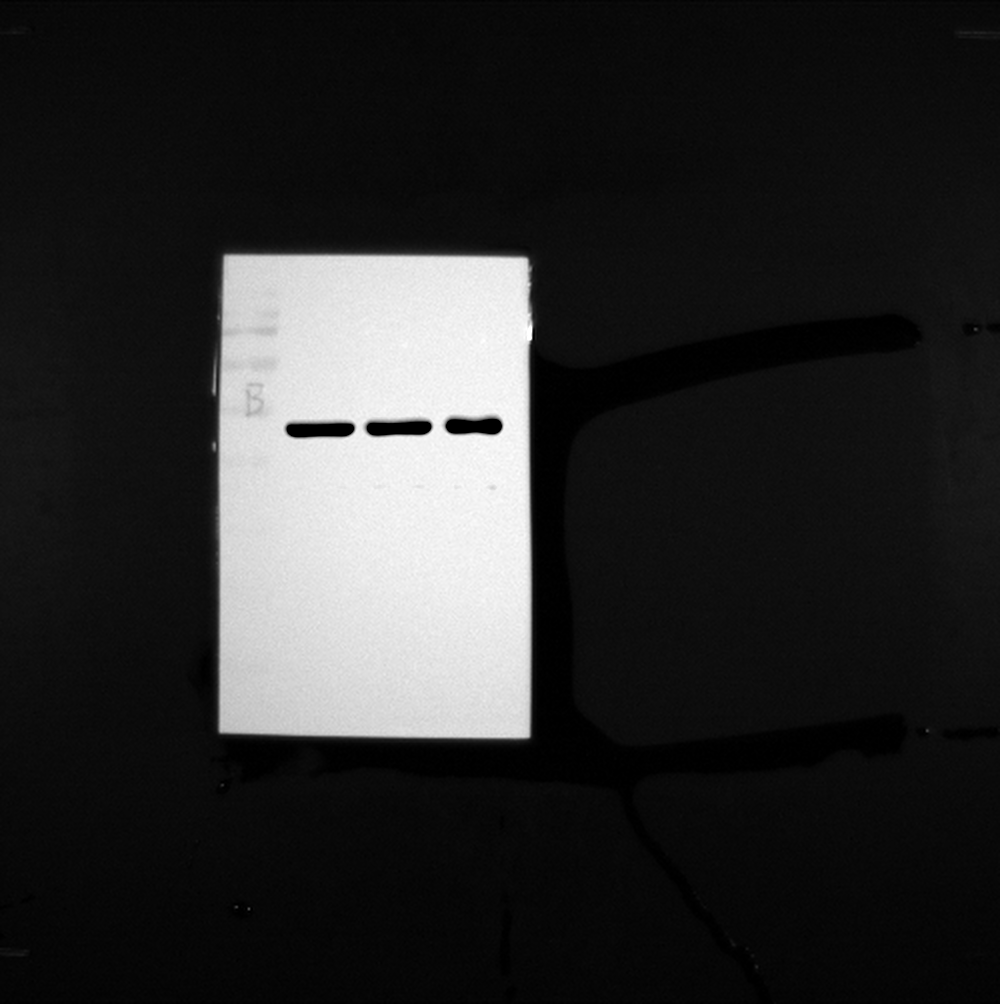

Supplement: Supplementary file 1 [file biology-14-00862-s001.zip › Cyclin E2-β actin.tif]

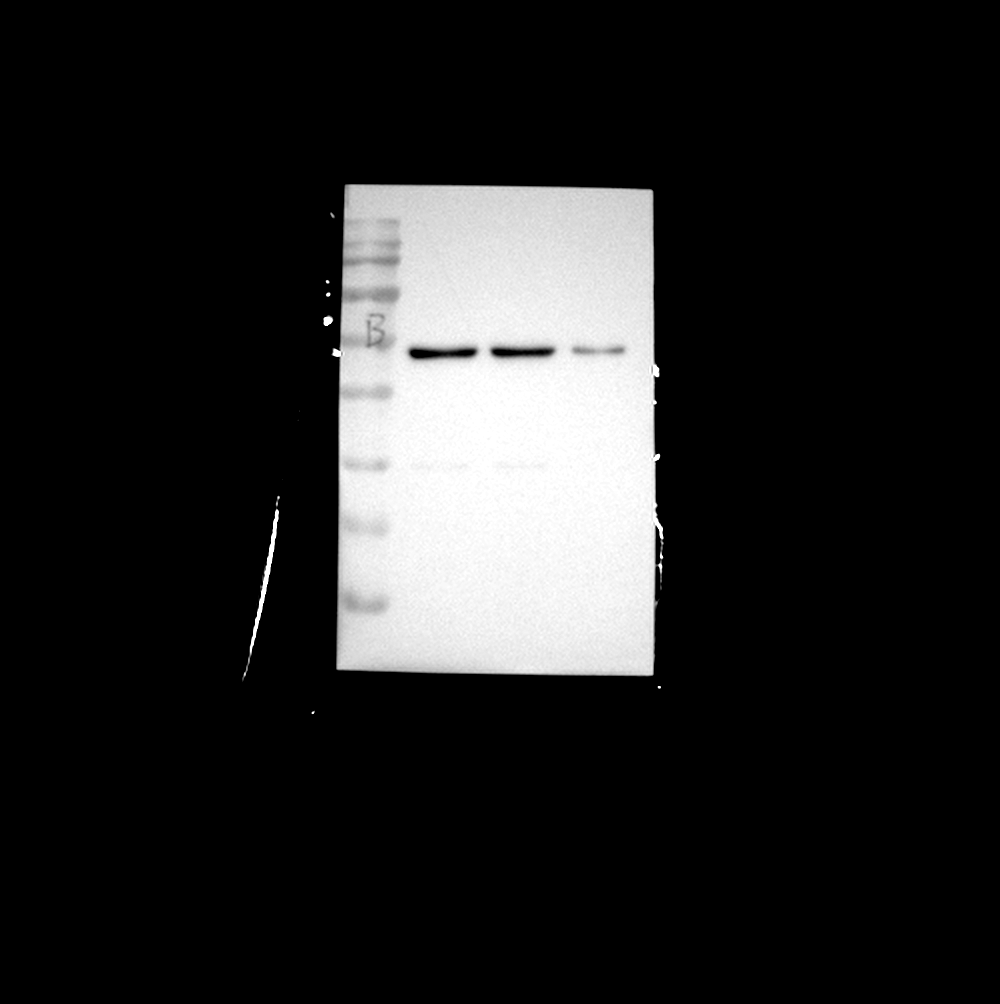

Supplement: Supplementary file 1 [file biology-14-00862-s001.zip › Cyclin E2.tif]
